# Hepatitis C Variability, Patterns of Resistance, and Impact on Therapy

**DOI:** 10.1155/2012/267483

**Published:** 2012-07-19

**Authors:** Cristina Simona Strahotin, Michael Babich

**Affiliations:** Division of Gastroenterology, West Penn Allegheny Health System, 1307 Federal Street, Ste 301, Pittsburgh, PA 15212, USA

## Abstract

Hepatitis C (HCV), a leading cause of chronic liver disease, cirrhosis, and hepatocellular carcinoma, is the most common indication for liver transplantation in the United States. Although annual incidence of infection has declined since the 1980s, aging of the currently infected population is expected to result in an increase in HCV burden. HCV is prone to develop resistance to antiviral drugs, and despite considerable efforts to understand the virus for effective treatments, our knowledge remains incomplete. This paper reviews HCV resistance mechanisms, the traditional treatment with and the new standard of care for hepatitis C treatment. Although these new treatments remain PEG-IFN-**α**- and ribavirin-based, they add one of the newly FDA approved direct antiviral agents, telaprevir or boceprevir. This new “triple therapy” has resulted in greater viral cure rates, although treatment failure remains a possibility. The future may belong to nucleoside/nucleotide analogues, non-nucleoside RNA-dependent RNA polymerase inhibitors, or cyclophilin inhibitors, and the treatment of HCV may ultimately parallel that of HIV. However, research should focus not only on effective treatments, but also on the development of a HCV vaccine, as this may prove to be the most cost-effective method of eradicating this disease.

## 1. Introduction: Burden of Hepatitis C

Hepatitis C (HCV) is a leading cause of chronic liver disease, cirrhosis, and hepatocellular carcinoma, as well as the most common indication for liver transplantation in the United States. The annual incidence of infection in the USA has declined from about 230,000 cases per year in the 1980s to an estimated 17,000 cases in 2007 [1, 2]. This decline has been largely attributed to changes in injection practices motivated by a concern for human immunodeficiency virus (HIV) risk [3]. Approximately 3.2 million persons have chronic HCV infection in the United States; however, the reservoir of chronically infected persons is still estimated at approximately 2.35%, representing approximately 160 million worldwide infected individuals [4]. Aging of the currently infected population is expected to result in an increase in the burden of hepatitis C in the next decade [5]. During that period, the number of HCV-related cirrhosis cases is estimated to increase by 31% and that of hepatocellular carcinoma (HCC) cases is estimated to increase by approximately 50% [5]. Estimates of hepatitis C prevalence range from <0.5% in very low endemic countries (e.g., northern European countries) to staggering rates of approximately 20% in highly endemic areas, including urban centers and the Nile Delta in Egypt [6]. 

## 2. HCV Virus

HCV, like hepatitis B virus (HBV) and HIV, is prone to develop resistance to antiviral drugs. Viral dynamics include daily virion production of 10^12^ with a half-life of 2-3 hours for free virions and less for intracellular virions. It has a very rapid mutation rate, with 2 error-prone viral polymerases that lack proofreading, and no overlapping reading frames, which make it prone to developing resistance. However, given the moderate infected cell turnover and the absence of a viral reservoir, or in other words, the lack of host genome integration or episomal persistence in infected cells [7], HCV has the full potential for eradication.

Hepatitis C is a flavivirus (of which yellow fever is the prototype) from the genus *Hepacivirus*. An HCV particle consists of a core of genetic material (RNA), surrounded by an icosahedral protective shell of protein, and is further encased in a lipid envelope. Two viral envelope glycoproteins, E1 and E2, are embedded in the lipid envelope [8]. The virus particle diameter is approximately 30–60 nm. The genome of 9,600 bases codes for ten proteins, though HCV isolates from different parts of the world differ in their length [9]. The 5′ and 3′ ends of the RNA are not translated into proteins (UTR) but are important to translation and replication of the viral RNA. The 5′ UTR has a ribosome binding site [10] (IRES—Internal ribosome entry site) that starts the translation of a very long protein containing about 3,000 amino acids (the polyprotein). The large polyprotein is later cut by cellular and viral proteases into the 10 smaller proteins that allow viral replication within the host cell or assembly into the mature viral particles [11].

Structural proteins made by the hepatitis C virus include the core protein E1 and E2, while nonstructural proteins include NS2, NS4, NS4A, NS4B, NS5A, and NS5B. 

The core protein has 191 amino acids and can be divided into three domains based on hydrophobicity: domain 1 contains mainly basic residues with two short hydrophobic regions; domain 2 is less basic and more hydrophobic and its C-terminus is at the end of p21; domain 3 is highly hydrophobic and acts as a signal sequence for E1 envelope protein. Both envelope proteins (E1 and E2) are highly glycosylated and important in cell entry. E1 serves as the fusing subunit and E2 acts as the receptor binding protein.

NS2 protein is a 21–23 kilo Dalton (kDa) transmembrane protein with protease activity.

NS3 is 67 kDa protein with serine protease activity at the N-terminal end and NTPase/helicase activity at the C-terminal end. It is located within the endoplasmic reticulum and forms a heterodimeric complex with NS4A, a 54 amino acid membrane protein that acts as a cofactor of the proteinase.

NS4B is a small (27 kDa) hydrophobic integral membrane protein with 4 transmembrane domains. Like NS3, it is located within the endoplasmic reticulum and plays an important role in recruitment of other viral proteins. It induces morphological changes in the endoplasmic reticulum forming a structure called the “membranous web.” NS5A is a hydrophilic phosphoprotein, which plays an important role in viral replication, modulation of cell signaling pathways, and the interferon response. The NS5B protein (65 kDa) is the viral RNA-dependent RNA polymerase.

Despite considerable efforts made to understand the basic structure and function of the virus, and the importance of this understanding for the development of antiviral treatment, our knowledge is far from complete. The exact mechanisms of HCV entry into hepatocytes have not yet been fully understood. Potential entry pathways into host cells may occur through complex interactions between virions and cell-surface molecules, such as CD81, LDL receptor, SR-BI, DC-SIGN, Claudin-1, and Occludin [12, 13], and ultimately via receptor-mediated endocytosis. Fusion of the virion envelope with cellular membranes delivers the nucleocapsid to the cytoplasm. After decapsidation, translation of the viral genome occurs in the cytoplasm, leading to the production of a precursor polyprotein, which is then cleaved by both cellular and viral proteases into three structural (virion-associated) and seven nonstructural (NS) proteins as discussed above. Via NS proteins, the viral genome attaches to the RNA replication complex, which is associated with rearranged cytoplasmic membranes. RNA replication takes place via the viral RNA-dependent RNA polymerase (RdRp) NS5B, which produces a negative strand RNA intermediate. The negative strand RNA then serves as a template for the production of new positive strand viral genomes. New genomes can then be translated, further replicated or packaged within new virus particles. New virus particles are thought to bud into the secretory pathway and are released at the cell surface. Release from the hepatocyte may involve the very low density lipoprotein secretory pathway [14]. 

## 3. HCV Resistance and Mechanisms of Action of IFN-*α* and Ribavirin

HCV exists as a mixture of populations of genetically distinct but closely related virions in every patient, including potentially drug-resistant variants that are present when antiviral therapy is initiated, thus conferring a quasispecies distribution. However, given its intracytoplasmatic replication and lack of intranuclear replication, there is no known potential for intracellular persistence [15, 16]. Drug-resistant variants often show reduced “replication fitness,” are undetectable with current technology, and have not gained much attention prior to development of the new direct acting antivirals (DAAs) [17, 18]. More sensitive techniques, such as ultra-deep pyrosequencing have been used to identify resistant variants prior to treatment, but these are not routinely used in current clinical practice [19–21]. Potent antiviral therapy eliminates sensitive strains, while resistant variants are uncovered and can expand. For many years, the recommended standard of care therapy for chronic HCV remained a combination of pegylated alpha-interferon (IFN-*α*) and ribavirin; however, neither drug exerts viral pressure. In other words, treatment failure is not due to selection of IFN-*α* or ribavirin-resistant variants but is more likely to occur due to inherent host factors (such as the presence of certain single nucleotide polymorphisms (SNPs) upstream of the IL-28B locus that correlate with the rate of SVR), inappropriate drug regimens and viral factors. Interferons are cellular proteins able to induce an antiviral state in their target cells, as well as cytokine secretion, recruitment of immune cells, and cell differentiation. Their metabolism and mechanisms were recently reviewed [22]. 

Immediately after injection, IFN-*α* binds to receptors present on various cells including hepatocytes, triggering a sequence of intracellular reactions that activate IFN-inducible genes (ISGs). The products of these genes are responsible for the IFN-*α*-mediated antiviral effects, achieved via two mechanisms. One is the induction of a nonvirus-specific replication inhibition in infected cells. IFN-*α* was found to directly inhibit HCV replication in vitro in the subgenomic replicon (a synthetic replication system using HCV nonstructural proteins in various cell lines) [23]. The second mechanism involves immunomodulatory effects that enhance the host's specific antiviral immune response, thus clearing the infected cells [22]. The induction of the antiviral state could potentially extend to noninfected cells, thus reducing the chance that they will become infected. Upon the interaction of IFN-*α* with its receptor, many complex effects are generated, including the induction of major histocompatibility complex class I (MHC I) antigen expression, activation of macrophages, natural killer cells and T lymphocytes, production of primarily T-helper 1 (Th1) cells, and decreased production of T helper-2 (Th2) cells. PEG-IFN-*α* also interacts with cytokines such as CCL chemokines and tumor necrosis factor (TNF)-*α*. Soluble TNF-*α* receptors (sTNF-R), which are released by activated neutrophils, mononuclear blood cells, and fibroblasts [24, 25] in response to mediators, such as interferon and TNF-*α* itself [25–28], retain their ability to bind circulating TNF-*α* and are important in regulating its activity. These sTNF-R may contribute to the anti-inflammatory action of IFN-*α*. All of these effects suggest that IFN-*α* simply accelerates the host immune response, though no studies have clearly proven it. 

HCV genotypes 1 and 4 are intrinsically more resistant to IFN-*α* than genotypes 2 and 3. More importantly, the sensitivity to IFN-*α* varies within each genotype. Consequently, the clearance of infected cells in patient who are interferon responders occurs much slower in genotypes 1 and 4, as compared to 2 and 3 [29, 30]. The mechanisms underlying these differences are yet to be defined. The combination of IFN-induced proteins and pathways responsible for inducing an antiviral state has not been completely mapped out, though various effectors have been proposed including the 2′–5′ oligoadenylate synthetase (2′–5′ OAS) system, Mx proteins, double-strand-RNA-dependent protein kinase (PKR), as well as other, less well-characterized/unknown IFN-induced intracellular pathways [22].

Ribavirin is a synthetic guanosine analog that undergoes intracellular phosphorylation, with the ultimate product, ribavirin triphosphate, being responsible for ribavirin's effects. *In vivo*, ribavirin has a moderate (<0.5 log HCV RNA reduction) and transient inhibitory effect of only 2-3 days duration on HCV replication, and this is only seen in about 50% of patients [31]. These effects are consistent with those seen in *in vitro* studies, and the modesty of the effect could be related to the drug's weak inhibitory action on the RNA-dependent RNA polymerase (RdRp) [32]. Fortunately, this effect is too weak and transient to account for selecting viral resistance to ribavirin. Despite its apparently weak antiviral effects, ribavirin remains essential to HCV treatment, as it appears to accelerate the clearance of infected cells through unknown mechanisms and to prevent viral breakthrough during treatment, and relapses following treatment, in patients on IFN-*α* [33]. Studies have suggested that ribavirin is an RNA mutagen, causing loss of viral “fitness” via lethal nucleoside accumulation during replication [34], though no excessive mutagenesis was noted during ribavirin therapy in HCV infection [35, 36]. Ribavirin, much like IFN-*α*, has immunomodulatory effects, including preferentially driving the immune system to produce more Th1 cells relative to Th2 [37]. Some reports have suggested that ribavirin can also amplify the intracellular IFN-*α* responses, via unknown mechanisms [38].

## 4. HCV Clearance

According to Tang et al., IFN-*α* attachment to interferon alpha receptors (IFNAR) [39] and the very rapid activation of Interferon Stimulated Genes (ISG) after interferon alpha administration [40] could explain the effect of IFN-*α* as a potent immunostimulant of an innate response in the first few hours and an adaptive response after the first four weeks. [41]. The degree of the ISG-induced innate response may result in a rapid decline of HCV replication (as measured by a decrease of HCV-RNA levels), which, if significant enough, causes very distinct CD4 and CD8 responses (shifting the immune response from innate to adaptive). Clinically, this phenomenon defines a group of patients known as rapid responders (HCV RNA serologic negativity achieved by week 4 of treatment) and differentiates them from patients with a less vigorous early reduction in viral load, known as slow responders. 

Sustained virologic response (SVR) is achieved when no virus is detected in the blood for six months after finishing treatment and prognosticates a 99% chance of indefinitely eradicating HCV, which for many experts is equivalent to curing HCV. Several events need to work in concert in order to achieve SVR, including (1) successfully achieve a rapid phase 1 effect by turning off viral replication, (2) effectively suppress the viral load throughout treatment, and (3) induce a solid and persistent Phase 2 effect [42].

It has been hypothesized that phase 2 is driven by the host's adaptive immune response in the context of sustained inhibition of virus production, while restoration of the innate immune response by viral inhibition leads to clearance of residual HCV-infected cells [43].

Both IFN-*α* and direct antiviral agents (DAAs) have very potent antiviral abilities and induce a very strong phase 1 response. Ribavirin is needed to prevent viral breakthrough during treatment and relapses after treatment, in patients who respond to the antiviral effect of IFN-*α* [33], and it appears to enhance the clearance of any residual infected cells, through unknown mechanisms, in patients treated with IFN-*α* combined with ribavirin or when the two are combined with DAAs [44]. Therefore, in addition to achieving a vigorous Phase 1 effect via the administration of IFN-*α*, use of ribavirin and DAAs may produce a similar vigorous Phase 1 as well as a gradual yet vigorous second phase, which combined with long-term treatment could ensure cure. Without a sufficient duration of treatment to promote the Phase 2 effect until all infected cells have been cleared, HCV replication will resume shortly after treatment completion and patients will experience a relapse. 

## 5. HCV Resistance to Specific Viral Inhibitors (Direct Antiviral Agents [DAAs])

HCV is prone to the development of resistance to specific antiviral inhibitors due to the quasispecies nature of the virus, its rapid dynamic, and the double error-prone RdRp [15], all leading to the development and/or persistence of drug-resistant mutants. It has been proposed that all single nucleotide-mutant drug-resistant viruses and all combinations of double-nucleotide mutant viruses preexist before treatment in most patients [45].

Based on experience with HIV and combined antiretroviral therapy (cART), it appears that the long-standing administration of specific viral inhibitors leads to formation of viral variants carrying amino acid substitutions that alter the original drug target, therefore, diminishing the therapeutic effects of the given drug. Similar substitutions present in a preexistent viral population may also allow those viruses to continue to replicate at a significant level during drug treatment. This phenomena may allow further mutations to accumulate, thus imparting a *partial resistance pattern*. Additionally, *compensatory mutations* may restore the viral capability to replicate at near the pretreatment stage [46]. *Resistance* is associated with a pattern where amino acid substitution confers a high level of drug resistance without diminishing the viral fitness in the presence of the drug. Cross-resistance between medications targeting the same site/function occurs both *in vitro* and *in vivo *and appears to be mediated by amino acid substitutions that confer reduced susceptibility to both drugs. The ability of viruses to replicate irrespective of the presence or absence of a drug has huge therapeutic implications, as it will lead to further evolution of the viral population. In theory, further evolution can result in a more fit, drug-resistant viral population that may remain in a patient, even in the absence of drug pressure. This further evolution should be preventable by quickly discontinuing the antiviral drug when a patient has a confirmed increase in HCV RNA levels while adhering to therapy, implying development of resistance. It is extremely important that clinicians adhere to the “futility rules,” which are defined below, to limit the development of viral resistance.

Resistance can be measured *in vitro* and *in vivo. In vitro, *resistance is measured on cell-free enzyme assays as the drug concentration needed to inhibit viral replication. Effective concentration (EC) defines the drug concentration required to inhibit the viral replication by 50% or 90% (E50 or E90). Less susceptible (hence more resistant) viruses will require more drug to be inhibited, thus are associated with an increase in E50 or E90. There is no consensus on what level of increase is needed to conclude that a given amino acid substitution confers resistance. In addition, the results *in vitro *must be cautiously interpreted in the clinical context because some viral variants with low-level resistance in vitro may be more aggressive in vivo than variants with higher-level resistance [47]. *In vivo, *resistance is influenced by genetic factors, the fitness of the virus, and drug exposure and is measured by viral load while on therapy. The best determinant of replication *in vivo *appears to be the fitness of the resistant variant [48] as a poorly fit virus is less clinically significant despite its being highly resistant, unlike a less resistant but fitter virus. Viral fitness implies the relative capacity of a viral variant to replicate in a given environment. Resistance mutations frequently compromise viral function and thus reduce viral fitness compared to wild-type virus in a drug-free environment. 

HCV has a relatively low genetic barrier to development of resistance to NS3/4A protease inhibitors. The genetic barrier refers to the number and type of nucleotide changes required for a virus to acquire clinical resistance to an antiviral agent [45]. Low genetic barrier to resistance implies that a single substitution can confer high resistance, while high genetic barrier to resistance implies that at least 3 substitutions are required to confer resistance. The protease inhibitors are able to select resistant variants *in vitro* based upon presence or development of amino acid substitutions. These substitutions appear to be located near the NS3 protease catalytic triad. These mutant variants render protease inhibitors unable to prevent viral polyprotein processing and, therefore, allow continued generation of mature viral proteins even in the presence of this class of drug [49–57].

Boceprevir and telaprevir are two recently developed NS3/4A protease inhibitor drugs.

Boceprevir- and telaprevir-based triple therapies are approved for the treatment of chronic hepatitis C, in combination with interferon-alpha and ribavirin, in the United States and Europe, where they are marketed under the brand names Victrelis and Incivek (Incivo in Europe), respectively. These drugs have opened up a new era in HCV therapy. They also illustrate quite well the significance of resistance. They share significant cross-resistance *in vitro, *with any given substitution producing different levels of resistance to the two drugs [52]. Most available data is derived from studies performed on patients treated with telaprevir (a reversible, selective, orally bioavailable inhibitor of the HCV NS3/4A serine protease) used in combination with IFN and ribavirin. Based on the level of drug present in serum, telaprevir is able to select resistant viral populations within days or weeks. The most resistant but least fit variants (with substitution at the 156 position) are selected early during therapy, while later they are quickly replaced by fitter variants carrying substitutions at various positions including 155, 36 and 155, 36, and 156 at the time of breakthrough [58, 59].

### 5.1. Triple Therapy with Telaprevir

At baseline, the main variants conferring resistance to telaprevir in patients with HCV genotype 1a (R155K, V36M) were detectable in 0.6–1.2% by population-based sequencing. In genotype 1b, variants conferring resistance to telaprevir (T54A, V36A, and A156T) were either undetectable, or detectable in a very small percentage of patients (0.07% for A156S, V36M and 2.1% for T54S) [60].

On-treatment virologic failure rates, defined as futility rules having been met (“futility rules” are defined below in the paper), were found to differ for HCV subtype 1a versus 1b and were also dependent on the prior treatment status of the patients. In treatment-naive patients, virologic failure was observed in 10% of patients with subtype 1a HCV and 3% of patients with subtype 1b HCV. Among previous treatment nonresponders, the on-treatment virologic failure rate was 38% and was again found to be higher in patients with subtype 1a than with subtype 1b. Finally, the on-treatment virologic failure rate for prior treatment relapsers was low at 10% [61, 62].

Population and clonal amino acid analysis of HCV species from patients who develop protease inhibitor resistance indicate that drug-resistant viral populations disappear in time, as illustrated by the follow-up studies for telaprevir. For genotype 1a HCV, the median time to lose resistant variants V36M and R155K was approximately 6 and 10 months, respectively, whereas loss of both mutations required approximately 13 months. For patients with subtype 1b HCV, the median half-life of resistant variants was shorter at 3 months for the common variants T54A, V36A, and A156T and 9 months for the more rarely occurring A156S variant [63]. Additional long-term followup, with a median duration of 29 months (range, 7 to 49 months) after treatment failure, showed that resistant variants were no longer detected in 85% of patients, and the rate of disappearance of resistant variants again depended on the specific mutation(s) (Table 1) [64].

### 5.2. Triple Therapy with Boceprevir

At baseline, the main observed variants conferring resistance to boceprevir in patients with HCV genotype 1a include V36M, T54S, R155K, which varies from the typical genotype 1b resistance profile, T54A, V55A, A156S, I/V170A, both of which occur with an overall low frequency [65]. As with telaprevir, viral drug resistance is more common at baseline and develops more frequently in subtype 1a than 1b, as subtype 1a HCV virus has a lower genetic barrier to resistance. Thus, for the most prevalent resistance variant, R155K, only 1 nucleotide exchange is required for subtype 1a HCV to develop drug resistance, whereas 2 exchanges are needed to generate the same resistance for subtype 1b HCV. Conversely, phenotypic resistance analysis in the HCV replicon [50, 52, 53, 66] showed that resistance-associated variants found in patients with subtype 1b HCV awarded a greater degree of resistance, with a 5- to 16-fold loss of susceptibility to boceprevir, than that found in patients with subtype 1a, with only 2- to 4-fold loss of susceptibility.

Current data confirm that the overall sustained virologic response (SVR) rate was lower in patients infected with HCV subtype 1a (53% to 64%) than in those infected with HCV subtype 1b (66% to 73%), again likely due to lower genetic barrier to resistance for subtype 1a. The likelihood of achieving SVR for genotype 1 prior treatment nonresponders was around 60% with boceprevir (combined with pegylated interferon/ribavirin) with an on-treatment failure rate of 40%, which is comparable to teleprevir. For treatment-naive patients, SVR rates approached 70–75% with boceprevir combination therapy, with a virologic failure rate of 20%–25%. For relapsers, the SVR reaches 75%, thus conferring an on-treatment virologic failure rate of 25% [67–70].

Long-term follow-up data on resistance-associated variants selected in non-SVR patients while on treatment showed that only approximately 20% of patients still had resistant variants detectable by population-based sequencing at 6–14 month followup [71]. The disappearance rate for the different NS3 protease resistance mutations was variable and is shown in Table 1.

## 6. Treatment Failure in Triple Therapy and Clinical Implications

Resistance is not an “all or none phenomenon.” Clinically significant resistance is usually associated with an “escape” pattern [72], where viral replication recovers quickly to pretreatment levels while amino acid substitution confers a high level of drug resistance without impairing fitness in the presence of the drug. If the virus is not very fit, the viral replication process will resume more gradually [46, 73–75]. Clinical resistance occurs if drugs levels are not sufficient to inhibit viral replication, and highly resistant viruses may need very high drug levels to inhibit their replication, which may not be achievable within acceptable safety parameters. In addition, sufficient drug trough levels must be maintained over time to achieve long-term viral suppression. Antiviral efficacy *in vivo* may remain stable if resistant variants replicate at low level and/or if the drug retains partial efficacy. Various patterns of HCV treatment failure have been reported, including viral nonresponse (persistent HCV RNA positivity on treatment), viral breakthrough on treatment, and viral relapse after treatment completion. In compliant patients, failure to respond to triple therapy derives mostly from lack of response to IFN-*α* and ribavirin, and consequent selection of preexistent viral species inherently resistant to DAAs. Conversely, treatment failure in noncompliant patients frequently results from de novo generation of viral mutants resistant to DAAs. This latter phenomenon is thought to account for most cases of viral breakthrough and relapse. 

As noted above, the failure rates in treatment-naïve patients are 20%–30% on triple therapy. In previously treated patients, the failure rates range as high as 50%–60%. Development of persistence of viral resistance depends in part on several host and treatment-related variables [61, 62, 69, 70]. Failure rates are higher in populations with patients with less favorable genetic background (IL-28 phenotypes CT or TT), including the African-American population, prior treatment nonresponders, HIV- or HBV-coinfected patients, post-liver-transplant patients, noncompliant patients, and patients with advanced fibrosis/cirrhosis. Multiple studies, including SPRINT 1- and -2, RESPOND-2, PROVE, REALIZE, and ADVANCE [67, 68, 76–79] have shown that the final treatment outcome with inclusion of DAAs was very much dependent on the degree of viral responsiveness seen during the first course of therapy with IFN-*α* and Ribavirin, a variable that likely reflects genetic host factors such as IL-28B genotype.

Certain single nucleotide polymorphisms (SNPs) upstream of the IL-28B locus correlate with the rate of SVR in patients treated with PEG-IFN-*α* and ribavirin, and recent data reveals a similar, though much less discrepant correlation in patients treated with triple therapy (Table 2).

Given the concerning data regarding development and persistence of drug-resistant mutants and on-treatment clinical resistance (data reviewed earlier in this paper), strategies should be employed to predict triple therapy failure early by monitoring viral kinetics. On-treatment “futility rules” exist and should be followed strictly. These rules define conditions that, if met, mandate immediate treatment discontinuation and are based upon viral kinetics. Failure to follow these rules could contribute to the development and persistence of more fit drug-resistant viruses, the presence of which, in turn, could jeopardize the patient's future chances to respond to newer antiviral treatments. Telaprevir is taken, with PEG-IFN-*α* and ribavirin, at a dose of 750 mg every eight hours for the first 12 weeks of the treatment. If HCV RNA is equal to or greater than 1000 IU/mL at weeks 4 or 12, virologic failure has occurred and triple therapy should be discontinued immediately. If the virus is detected to any degree at week 24, dual therapy should also be discontinued at that point, as the likelihood of achieving SVR is very low. When using boceprevir, the treatment algorithm is different and involves a 4-week period of PEG-INF-*α* and ribavirin dual therapy, also known as the “lead in” period, followed by triple therapy with addition of 800 mg of boceprevir every eight hours for a variable period of time; PEG-IFN-*α* and ribavirin will continue until treatment completion. A viral load equal to or greater than 100 IU/mL at week 12 is equivalent to virologic failure, and all three drugs should be stopped to avoid resistance. Alternatively, any detectable viral load at week 24 again implies again lack of response, and treatment should be terminated.

Failing triple therapy raises concerns regarding possible adverse consequences for the liver disease itself and also for the potential to respond to future DAA-based therapy. The rate of HCV replication ultimately returns to the pretreatment stage following treatment failure. Consequently, the disease appears to resume its progression to cirrhosis at the same pretreatment rate. As HCV is not a cytopathic virus, most of the hepatocellular damage is caused by the host immune system targeting the persistently infected hepatocytes. In terms of impact for future therapy, one can hypothesize that if protease inhibitor-resistant viruses, which have acquired greater fitness during therapy with DAA's, remain the dominant viral population within a host then, at the time of retreatment, the use of medications with cross-resistance with telaprevir and boceprevir would not be expected to work. Patients failing triple therapy should be advised that many new HCV drugs are under development and may become available outside study protocols within several years, but it is yet to be defined whether these patients will be appropriate candidates for retreatment with these drugs. These patients must continue to be clinically monitored for signs of disease progression.

## 7. Conclusions

A new standard of care for treating genotype 1 hepatitis C infected patients is now available, for both treatment-naïve patients and treatment-experienced patients. This treatment remains PEG-IFN-*α* and ribavirin-based but adds either telaprevir or boceprevir, protease inhibitors that became the first DAA drugs specifically targeting the HCV NS3/4A protease, and that were approved by the FDA in May, 2011. This new “triple therapy” has allowed patients to achieve dramatically greater rates of viral cure than previously, but treatment failure remains a possibility. Failure to achieve a viral cure with this regimen most likely results from the low viral genetic barrier to resistance to the protease inhibitors, allowing *de novo* formation of drug-resistant viral mutants, superimposed on a weak host response to pegylated IFN-*α* allowing persistence of drug-resistant viruses that were present at baseline. Treatment failure would allow continued progression of the liver disease and may affect candidacy for and/or responsiveness to the next line of DAAs in development. The future may belong to agents such as nucleoside/nucleotide analogues, nonnucleoside RNA-dependent RNA polymerase inhibitors, or cyclophilin inhibitors, and the treatment of hepatitis C may ultimately parallel that of HIV in the near future, with use of various combinations of “drug cocktails.” 

The need for a vigorous host response to IFN in order to achieve a viral cure with current therapies, and patients' failure to achieve such a response, or intolerant of IFN-related side effects, has led many investigators to develop drugs that could provide an IFN-free curative regimen. IFN-free regimens are no longer a dream, but a reality that may be available in the clinic in the next 5 years. It is possible that some of these regimens will also be RBV-free. PSI-7977, now GS-7977, a potent uridine nucleotide analog in Phase 3 development, demonstrated >90% SVR rates in the PROTON trial, where it was used in combination with PEG/RBV to treat patients infected with HCV GT1, and 100% SVR rates were achieved in the ELECTRON trial when used to treat patients infected with HCV GT2/3 with an interferon-free regimen using only PSI-7977/RBV. These high SVRs are attributable to PSI-7977's antiviral potency, a lack of detectable preexisting resistant HCV variants, and excellent safety and tolerability profile [82]. Another 12-week, interferon-free combination of two experimental, once-daily drugs (ABT-450, a hepatitis C protease inhibitor that requires blood-level boosting with the HIV protease inhibitor Norvir, and ABT-072, an HCV nonnucleoside polymerase inhibitor) plus ribavirin achieved SVR at 12 weeks in 91% of noncirrhotic first-time treatment takers with HCV genotype 1 and an IL-28B CC genotype, according to data from the PILOT trial, presented on April 19, at the 47th Annual Meeting of the European Association for the Study of the Liver (EASL) in Barcelona [83]. Researchers at EASL also presented data from the COPILOT trial, an interferon-free clinical trial combining ribavirin, Norvir-boosted ABT-450 and ABT-333 (a twice-daily HCV non-nucleoside polymerase inhibitor) in first time-treatment takers and null responders with HCV genotype 1. ABT-450 and ABT-333 maintained hepatitis C virus levels undetectable for 12 weeks after completing treatment in over 90% of first-time treatment takers and 47% of treatment experienced people [84]. Having more, and more effective, treatments should not, however, diminish our efforts to develop a hepatitis C vaccine, as the cost of treatment continues to increase, the currently available agents continue to carry the potential for severe side effects, and cure rates still fall short of the ideal goal of 100%.

## Figures and Tables

**Table 1:** Mutations conferring resistance to telaprevir.

HCV resistant variant	Patients without resistant variants at followup
T54A	94%
A156S	88%
V55A	86%
V36M	75%
R155K	68%
T54S	68%

**Table 2:** Role of IL-28B in HCV treatment.

IL-28B SNP	PR (IDEAL)		Telaprevir (advance)		Boceprevir (SPRINT-2)	
	ITT population	Adherent population	TVR/PR	PR control	BOC/PR^∗^	PR control
CC	69%	79%	90%	64%	80–82%	78%
CT	33%	38%	71%	25%	65–71%	28%
TT	27%	26%	73%	23%	55–59%	27%

^∗^Includes BOC/RGT and BOC/PR48 arms [61, 69, 80, 81].
